# What have we learnt from measles outbreaks in 3 English cities? A qualitative exploration of factors influencing vaccination uptake in Romanian and Roma Romanian communities

**DOI:** 10.1186/s12889-020-8454-x

**Published:** 2020-03-23

**Authors:** Sadie Bell, Vanessa Saliba, Mary Ramsay, Sandra Mounier-Jack

**Affiliations:** 1 Department of Global Health and Development, Faculty of Public Health and Policy, London School of Hygiene & Tropical Medicine, 15-17 Tavistock Place, London, WC1H 9SH UK; 2 Department of Immunisation, Hepatitis and Blood Safety, Public Health England, London, NW9 5EQ UK

**Keywords:** Vaccination, Measles, Romanian communities, Roma communities, England, Inequalities, Vaccination attitudes and behaviours, Vaccination access, Primary health care

## Abstract

**Background:**

Since 2016, large scale measles outbreaks have heavily affected countries across Europe. In England, laboratory confirmed measles cases increased almost four-fold between 2017 and 2018, from 259 to 966 cases.

Several of the 2017–18 measles outbreaks in England particularly affected Romanian and Roma Romanian communities, with the first outbreaks in these communities occurring in Birmingham, Leeds and Liverpool. This study explored factors influencing vaccination behaviours amongst Romanian and Roma Romanian communities in these three cities.

**Methods:**

Across Birmingham, Leeds and Liverpool, we conducted semi-structured interviews with 33 key providers to explore their experience in delivering vaccinations and managing the outbreak response. We also interviewed 9 Romanian women in one of the cities to explore their vaccination attitudes and behaviours. To categorise factors affecting vaccination we applied the 5As Taxonomy for Determinants of Vaccine Uptake (Access, Affordability, Awareness, Acceptance and Activation) during data analysis.

**Results:**

Factors related to access and acceptance, such as language and literacy, ease of registering with a general practice, and trust in health services, were reported as the main barriers to vaccination amongst the communities. Concerns around vaccination safety and importance were reported but these appeared to be less dominant contributing factors to vaccination uptake. The active decline of vaccinations amongst interviewed community members was linked to distrust in healthcare services, which were partly rooted in negative experiences of healthcare in Romania and the UK.

**Conclusion:**

Access and acceptance, dominant barriers to vaccination, can be improved through the building of trust with communities. To establish trust providers must find ways to connect with and develop a greater understanding of the communities they serve. To achieve this, cultural and linguistic barriers need to be addressed. Better provider-service user relationships are crucial to reducing vaccination inequalities and tackling broader disparities in health service access.

## Background

Measles is a highly contagious viral disease, associated with substantial levels of morbidity and mortality [1]. A goal within the European Vaccine Action Plan is to eliminate measles from the European Region by 2020 [2]. However, since 2016, following over a decade in which Europe experienced a decline in measles cases, large scale measles outbreaks have occurred across the region [3].

In 2018, there were 83,540 measles cases and 74 measles-related deaths in the World Health Organisation (WHO) European Region, compared to 5273 measles cases and 13 measles related deaths in 2016 [4]. England has also been hit by a sharp and persistent increase in measles cases, despite the two-dose MMR vaccination that protects against measles, mumps and rubella being provided freely on the National Health Service (NHS) through general practice (GP). Between 1st January 2018 and 31st December 2018, there were 966 laboratory confirmed measles cases in England, more than tripling from 259 during the whole of 2017 [5].

The European Centre for Disease Prevention and Control highlights that populations on the move within Europe are at risk of under-vaccination and therefore more susceptible to vaccine-preventable diseases. Factors that affect vaccination access amongst migrant populations include difficulties understanding and navigating different health systems, and overcoming cultural and linguistic barriers [6, 7].

Recent outbreaks in England have been linked to the travel of under-vaccinated people to measles endemic countries in Europe, and the subsequent transmission of measles in England to communities with poor vaccination coverage. These include people that may have missed vaccinations during the ‘Wakefield’ MMR scare, and other populations known to have lower vaccination coverage such as Traveller, migrant, the ultra-Orthodox Jewish, and Anthroposophic (Steiner) communities [8].

Several of the 2017–18 measles outbreaks in England specifically affected Romanian and Roma Romanian communities [8]. In 2017, there were just under 400,000 Romanian born residents living in the UK [9]. Although the number of Roma and their nationality is not accurately known, estimates indicate the total number of Roma in the UK to be at least 200,000 [10].

Although Gypsies, Roma and Travellers (GRT) are often considered collectively, due to similarities in their customs, beliefs, and experiences of marginalisation, each are a distinct ethnic group [11]. The UK’s Roma are mainly sedentary communities living in fixed accommodation. Roma in the UK are from Eastern and Central Europe, with origins in West India. Roma have a distinct language, Romani or Romanes, which contains many different dialects, and may speak the language of their nationality (e.g. Romanian, Czech, Slovak) as a second language.

Roma are one of the largest and most marginalised ethnic minority groups in Europe and have experienced an extensive history of discrimination, persecution, and social exclusion [12, 13]. In the UK, Roma experience inequalities in standards of living, education, employment, and health [14–18]. Romanian communities in the UK have also been found to experience health inequalities [19], and encounter discrimination [20].

As health datasets do not routinely record information by nationality or minority ethnic group, vaccination uptake amongst Romanian and Roma Romanian communities in the UK is not known. However, numerous studies across Europe have found vaccination uptake to be lower amongst Roma compared to non-Roma communities across Europe [21–23]. Vaccination uptake has also been reported as lower in Romania compared to other European countries but has not been examined amongst Romanian communities in the UK. In the UK, coverage of MMR first-dose at 12 months in 2018 was 92% and for MMR second-dose at 3 years and 4 months was 88%. In Romania, coverage of MMR first-dose at 12 months in 2018 was 90% and for MMR second-dose at 5 years was 81%, and there were around 1300 measles cases reported in Romania between March 2018 and February 2019 [24].

As Romanian and Roma communities are at risk of under-vaccination and were affected by measles outbreaks in 2017–18 in England, we conducted a qualitative interview study to explore factors contributing to vaccination uptake amongst these communities. Understanding these factors is crucially needed to effectively address inequalities in vaccination uptake and meet the health needs of these communities.

## Methods

Our study took place in Birmingham, Leeds and Liverpool (Table 1), cities that experienced measles outbreaks in 2017–18 that particularly affected Romanian and Roma Romanian communities.

**Table 1:** Outbreak size and duration, and MMR vaccination coverage in Birmingham, Leeds and Liverpool (2017–18)

	Outbreak size (confirmed cases) and duration	MMR 1st dose at 24 months (2017–18)	MMR 1st dose at 5 years (2017–18)	MMR 1st and 2nd dose at 5 years (2017–18)
Birmingham	116 cases (November 2017– June 2018)	87.6%	93.7%	81.6%
Leeds	36 cases (November 2017 to January 2018)	92.9%	95.8%	88.2%
Liverpool	22 cases (November 2017)	93.0%	95.0%	86.2%

We conducted semi-structured interviews with providers involved in vaccination delivery and outbreak management in each city, including frontline vaccinators and representatives from Public Health England (PHE) Health Protection Teams, Screening and Immunisation Teams and Local Authorities. We identified and approached providers through PHE Health Protection Teams in each city. The teams were able to link us with providers that they considered key in the outbreak response.

Written informed consent was obtained from all study participants. The interviews lasted 30–45 min and took place in person or via telephone. Providers were asked about their experiences in delivering vaccination services to Romanian and Roma service users.

To gain insight from the communities, we also conducted semi-structured interviews with Romanian community members (CMs) living in one of the cities. This deviated from our original proposal to interview CMs in each of the cities, due to difficulties in identifying and engaging with CMs within the timeframe of the study. CM recruitment took place through an Eastern European women’s community group led by a Gypsy, Roma and Traveller Outreach worker. Eligible CM participants included parents and grandparents. Interviews with CMs lasted approximately 30 min and were conducted face-to-face, with the assistance of a Romanian speaking female interpreter. During the interviews, we asked the CMs to talk about their vaccination experiences in relation to themselves and any children/grandchildren. CMs were compensated with a £10 gift voucher.

Interviews were transcribed verbatim and analysed thematically using the stages outlined by Braun and Clarke [25]. During theme generation, a matrix was created using the “5A’s Taxonomy for Determinants of Vaccine Uptake” to categorise factors associated with vaccine uptake [26]. The categories within the taxonomy are: Access, Affordability, Awareness, Acceptance and Activation [26] (Table 2). Contributing factors to vaccine uptake were classified in this way to identify where to target recommendations to improve uptake.

**Table 2:** 5As Taxonomy for Determinants of Vaccine Uptake

Factor	Definition
Access	The ability of individuals to be reached by, or to reach, recommended vaccines
Affordability	The ability of individuals to afford vaccination, both in terms of financial and non-financial costs (e.g. time)
Awareness	The degree to which individuals have knowledge of the need for, and availability of, recommended vaccines and their objective benefits and risks
Acceptance	The degree to which individuals accept, question or refuse vaccination
Activation	The degree to which individuals are nudged towards vaccination uptake

## Results

### Participants

Interviews were conducted with 33 providers and 9 CMs. The CMs were all women, and 3 of these women self-identified as Roma. Providers from a range of job roles were recruited from different organisations on the basis that they were involved in vaccination delivery to Romanian and Roma Romanian communities, or in an outbreak response. Participant demographic characteristics are reported in Tables 3 and 4.

**Table 3:** Providers

Organisation/job role	Number of interviews		
	Liverpool	Leeds	Birmingham
Public Health England – Health protection team	1	1	5
Screening and immunisation team member	1	2	1
Practice nurse	–	4	1
General practitioner	–	1	
GP practice manager	–	3	2
Council	1	–	4
School nurse	1	–	–
Community immunisation nursing team member	2	–	–
Social exclusion team member	2	–	–
Health visitor	–	–	1
**Total = 33**	**8**	**11**	**14**

**Table 4:** Community members

No.	Length of time living in the UK	Self-reported ethnicity and nationality	Children	Reported vaccination status of children	Where vaccinated
**1**	3 months	Romanian	4 children, age range 2–14 years.	Children fully vaccinated.	Romania and the UK
**2**	3 years	Romanian	5 children, age range 1.5–14 years	Children only vaccinated with BCG.	Romania and the UK
**3**	1 year	Romanian	No children	N/A	No recent vaccinations (all vaccinations in childhood). All vaccinations received in Romania.
**4**	1 year	Romanian	5 children, age range 5–16 years	Children fully vaccinated	Romania and the UK
**5**	3 years	Romanian – mother was Romanian and father was Roma Romanian	2 children, aged 7 months and 5 years	Children fully vaccinated	Romania and the UK
**6**	2 years	Roma Romanian	4 children, age range 1.5–13 years	Children fully vaccinated	Romania and the UK
**7**	3 years	Romanian	3 children, age range 6 months-3 years	Children fully vaccinated	Romania and the UK
**8**	2 years	Romanian	3 children, age range 3 to 9. Participant also pregnant at the time of interview.	Youngest child has not been vaccinated. Mother has also never had any vaccinations.	Romania and planned vaccinations for her youngest child in the UK
**9**	5 years	Roma Romanian	2 children, aged 19 and 21. One grandson aged 4 years.	2 vaccinations declined for grandson – flu and another vaccination.	Vaccinations for her children all received in Romania. Vaccinations for her grandson given in the UK.

Ten main factors were reported to influence vaccination uptake: primary care accessibility and acceptability, language and literacy, perceptions around vaccination costs, competing priorities to vaccination, awareness of vaccines and access to vaccine information, perceptions around measles severity and the benefits of vaccination, trust in the healthcare system and vaccines, and prompts to vaccinate. These factors are explored under the categories used in the “5A’s Taxonomy for Determinants of Vaccine Uptake” [26].

### Access

#### Primary care accessibility and acceptability

Providers considered access to primary healthcare to be a major barrier to vaccine uptake. In all 3 cities, providers reported that registration with general practice and lower primary care use were an issue amongst the communities. Lower usage of primary care by the communities was partly perceived as due to differences in health-seeking behaviours. Providers, particularly in Birmingham, noted that community members were more likely to access accident and emergency (A&E) services than primary care, and only then once they felt very unwell.

*‘in their country they’re not going to go to see the doctor, unless they’re very ill and they’re going to go straight to the hospital. You don’t go through the GP.’ (Provider 23)*

Several providers felt that the concept of primary care and preventing illness was often not adopted by community members, with one provider reporting: *‘they just don’t believe in any medical intervention as such …*. *it’s very low on their horizon and priority*.’ Other providers considered that uncertainties around entitlement to care prevented people from accessing health services until they became very unwell and realised that they could not self-manage their health.

In their engagement with the communities, providers found that navigating the health system was challenging and unclear for community members, particularly in the presence of language barriers. The process of registering with a general practice was not always clear. For instance, providers found that some community members were unaware of a need to register their new-born child at their GP practice, considering that this would be an automatic process if the mother was already registered there.

Experiences of discrimination were also not uncommon, specifically providers highlighted this in relation to encounters with GP receptionists.

*‘..to get to the GP you have to get past the front desk and it’s the front desks that we are finding are really resistant to registering patients, following up, etc … … So first of all you’ve got to get passed the front desk, and if you don’t speak English that’s damn near impossible, and some of the receptionists are like out and out rude to the new arrivals, some of them are extremely hostile to registering patients, particularly if they are from Eastern Europe.’ (Provider 8)*

Amongst the CMs we spoke with, registration with a general practice had been reported as relatively easy; however, this appeared to be because the CMs had been helped in the process by friends and family. Amongst the CMs, their experiences of accessing general practice were largely positive, although they were aware of friends and family members that had experienced inadequate care.

*‘my mum lives here in the UK … . but her general practitioner throws her out [of] the door every time she has problems because she can’t speak English, they’ve got her out during the appointment. They’ve done this three times already. They push her out. And she’s feeling really sick … . she’s afraid.’ (Community member 6)*

#### Language and literacy

CMs reported language and literacy as major barriers to accessing credible vaccine information and giving informed consent for vaccination. Providers also reported their awareness of these issues, and highlighted communication as a factor affecting their ability to properly explain vaccinations and to promote vaccination. The time-allotted to appointments with midwives and those working in general practice, reported as just 15 min, was considered unrealistic, particularly when trying to overcome communication barriers.

Providers often struggled to distinguish the difference between Roma and Romanian, particularly when it came to language. Many Roma speak Romani as a first language, and the language of their nationality may be their second language. Romani has many different dialects and providers highlighted that access to a professional Romani speaking interpreter was not possible. Even when providers were aware that Romanian was not the preferred language for community members, or one they were proficient in, they remained reliant on accessing Romanian interpreters due to a lack of professional Romani interpreters. The use of Romanian interpreters could also be problematic, given the history between these groups

*‘ … . some Roma communities feel very, apparently, badly treated by Romanians and therefore having a Romanian translator might not actually support understanding and translation. Well, [it] might introduce more problems and not solve the problem we’re trying to solve.’ (Provider 11)*

Most of the CMs that we spoke with were able to access a Romanian speaking telephone interpreter at their GP practice. However, in their experience, providers were concerned that using telephone interpreters was not always effective.

*‘In your appointment, it’s very hard to explain, with the language, what each illness is, and you sometimes wonder what the Language Line is actually saying, because you’re trusting their interpretation. Sometimes the patient looks totally confused with the Language Line. So, it’s a very hard job.’ (Provider 14)*

One CM also highlighted that those requiring an interpreter may not be aware that GPs are obligated to provide interpreting services. Instead they may seek their own interpreters, who may be potentially exploitative.

‘*there are a lot of dodgy people maybe on the internet who offer services, who offer to help you … and they charge a lot.’ (Community member 1)*

CMs particularly reported a lack of interpreters available to explain school-based vaccinations, and in one instance an online translation tool was being using by health visitors. One CM had experienced difficulties in understanding and completing the informed consent form for her child’s flu vaccination at school. Not being able to complete the form had meant that the child missed her flu vaccination at that time.

CMs also highlighted that literacy barriers may be an issue amongst the communities, and that written information (while useful) would not be accessible to everyone.

*‘it would be really helpful to have both - leaflets and some advice in person. With the leaflets is really hard because a lot of Romanians don’t go to school and can’t really read. So it’s probably better or more helpful if they, somebody could explain to them in person, face to face.’ (Community member 4)*

In order to try and manage communication barriers, some CMs discussed having translation apps on their phones, or attending appointments with their family members.

*‘she will try to go [to the school] with her daughter, 11 years-old, because she’s good at English, and maybe the daughter can help mum to understand everything about this immunization.’ (Community member 6)*

One CM also talked about receiving direct help from linking services at her local council to organise appointments.

### Affordability

#### Perceived financial costs

From their contact with community members, several providers reported a lack of clarity around payment for health services that could pose as a barrier to accessing healthcare and vaccination.

*‘ … . if you are new into the country there are language issues, you don’t know how to navigate the health system, how do you understand if you’re one of those migrants that will be charged or won’t be charged … .’ (Provider 1)*

#### Competing priorities

In the context of other competing demands, vaccination was often not one of the main priorities for community members. The communities were described as having a more reactive response, living day-by-day, and dealing with immediate stressors. Competing priorities related to financial instabilities.

*‘in the great scheme of things, vaccination tends to be a little bit lower down the list when you’re struggle even to wonder what you’re going to feed [your children], or how you’re going to live for the rest of the day’ (Provider 8)*

Given this context, booking vaccination appointments in advance was not considered to be particularly effective, indicating the benefits of using a different approach such as drop-in vaccination sessions.

*‘The biggest problem we think exist is if you send them pre-booked appointments, so if you get them to book an appointment that just does not work; they don’t live like that … .if you book them appointments, even if they’ve booked them themselves, it doesn’t work, the DNA (did not attend) rates are very high. [They are more likely to come] if you tell them come this morning … ..we’ve got a clinic coming like this morning, you can walk in. They understand that.’ (Provider 12)*

### Awareness

Given the language and literacy barriers experienced by CMs, being able to locate credible information about vaccines in translated forms was difficult. The majority of CMs that we spoke with were not provided with written vaccination in translated forms. Several sought their information from family and friends, in addition to healthcare professionals. One CM also discussed the use of social media, accessing online chats and searching for information on YouTube.

Amongst the CMs that we spoke with, there was an awareness around the vaccine schedule in the UK; however, providers reported awareness as an issue within the communities.

*‘I think there was misunderstandings or wrong levels of awareness. I think people were saying to the school imms service when they were trying to offer MMR vaccinations or when the health protection team were asking parents about when their kids had their MMR, they were being told yes, they had the injection when they were born, and they are all okay. We are not quite sure what that was. I think we were all doubtful that it was MMR. It may just have been something else.’ (Provider 11)*

One provider in Birmingham had also heard the belief from community members that ‘one shot cures all diseases’, highlighting what appeared to be a lack of awareness around the vaccine schedule and the need for different vaccines for protection.

### Acceptance

#### Perceptions around measles severity

Providers, particularly in Birmingham, reported that measles was not necessarily a disease that caused concern amongst the communities. Several providers believed that for some community members their children contracting measles was a ‘rite of passage’

*‘the thought was that it wasn’t particularly a disease that they [the Romanian communities] worried about, so I don't know whether it was the attitude to the vaccine or the attitude to the disease … ..I know that they appeared not to be worried enough to have the vaccine.’ (Provider 25)*

It was considered beneficial to contract measles rather than vaccinate, so as to develop a ‘natural immunity’ to the disease.

*‘In one family we had about eight members catch the virus, and they’ll say, “Well, it’s better to catch the natural infection than the injection.” Well, that isn’t always true, because you get a lot of symptoms, and we saw people in ITU and these were like young women, fit and healthy, and one ended up in ITU. She was really very ill, and could have died. We’ve had a couple of kids as well … . I think they just think, “Measles, it’s a bit of a rash and that’s it,” (Provider 15)*

#### Perceptions around the benefits of vaccination

Amongst the interviewed CMs, most considered vaccinations beneficial and important, particularly those that had witnessed vaccine-preventable diseases. CMs were often nervous ahead of their child’s first vaccination, but this passed with positive vaccination experiences. A minority of CMs had not fully vaccinated their children as they believed that vaccines could cause more harm than good, producing damaging side effects.

*‘I’m worried about illness and catching cold. Other children got sick afterwards, with swollen throats and then cancer and many other problems. Like lung diseases’ (Community member 2)*

*‘some children had one-week high temperature, some children because it was something with the nose, chocked and they couldn’t breathe.’ (Community member 9)*

Amongst the CMs that declined vaccination, there was also the belief that vaccinations are ineffective. This was discussed particularly in relation to the influenza and MMR vaccine.

*‘other people had the vaccine against this disease, measles as you call it, they had the vaccine and still got the disease. This makes me doubtful … ..‘I asked a nurse when I was in Romania. I asked her why do you have them vaccinations against measles if they don’t protect children and they still get the measles? And she said it happens for them to still get.’ (Community member 2)*

Another belief was that vaccinations were unnecessary, as their children were well without.

*‘I didn’t get the vaccines for my children neither in Romania nor here. None. I think it’s the best way. They are much better this way. They don’t catch cold, they don’t get ill.’ (Community member 2)*

Other beliefs that providers had noted within the communities, which were not raised during interviews with CMs, were that vaccines could causes impotence, that vaccines are part of a conspiracy theory by religious leaders, and that vaccines contain human tissue.

#### Trust in vaccinations and health services

Past experiences of vaccinations and health care, in Romania and the UK, affected the decision to access vaccinations and health services amongst some of the community members. Understandably, negative experiences could create a distrust and fear of vaccines and health services. This highlights the importance of understanding the context of people’s lives and how this shapes health decision-making.

*‘I was afraid to have the vaccination for the boy. I was afraid because in Romania a lot has happened, I got scared and I refused … . why did I refuse? Because someone in our village in Romania died because of the vaccination. The vaccine wasn’t done properly. I was afraid when the boy was born to have the vaccine on him. I refused the vaccination … .. the doctor no longer works there, he was put to trial. After the trial he was released from the hospital, they replaced him’ (Community member 8)*

Although it was unclear what exactly had happened to this child, the reporting of his death shortly after vaccination had generated a fear in the community. In this instance, the CM had become distrustful of doctors and subsequently concerned about vaccinations. This CM had also experienced other negative experiences of healthcare in Romania, in relation to herself and family members, which had formed her opinion that healthcare professionals are money-driven and cannot be trusted.

*‘In Romania, if you don’t have money they leave you to die at home.’ (Community member 8)*

In the UK, this CM had gone on to experience positive experiences of healthcare, which in turn had changed her attitude towards vaccinations.

*‘after [I] saw how the NHS is here in England, [I] changed my mind … . that’s why I wanted the vaccination for my son, because the doctors are different here … … .[I] built up a relationship n and confidence with professionals from NHS because [I was] in the hospital with the little one for a while one, when the little one was just 11 months old, and they saved his life’ (Community member 8)*

It was highlighted by providers that through discussion with community members trust could be developed and that this promoted vaccination uptake.

*‘ … once I think I get a bit of a conversation going with the Roma mums, they do let me in and they will let me immunise. They’re not against immunisations, they will let you immunise. That’s the big difference I find, but it’s that level of trust that you need to get with the Roma’ (Provider 20)*

### Activation

Providers found that their blanket approach for reaching service users, such as GPs sending vaccination reminders to CMs via letter or text message, was not a particularly effective way of reaching the communities, particularly the Roma. This was due to communication barriers, and the transiency within Roma communities.

*‘ … . this community really don't stay [anywhere] very long, so to even have an address is probably quite difficult … . they seem to move on, and it’s not the same people in houses from one week to the next.’ (Provider 25)*

Face-to-face communication was considered a much more effective approach to reaching communities and gaining their trust, using outreach strategies. In order to promote vaccination, although costly, providers also considered that it would be beneficial to involve members of the community as vaccine advocates. It was also felt that there needed to be a more integrated approach, involving different local organisations (e.g. schools, social care providers, local authorities, health visiting and midwifery services, and general practice) in identifying, understanding and building trust with communities.

## Discussion

We found that contributing factors associated with ethnicity and nationality, including language and literacy, cultural and historical backgrounds, and experiences of discrimination and social exclusion, influenced vaccination uptake in the communities.

Factors related to access and acceptance, such as language and literacy barriers, navigating and registering with primary care, and trust in health services, were considered the main barriers to vaccination. Many of these barriers are also shared by other migrant and minority groups, across different countries [27–29]. The funding within the Romanian health system is concentrated in secondary care, and focused on treatment rather than prevention, which may help to explain the reported lower primary healthcare use amongst Romanian communities [30].

In our study we found that several community member participants that had declined vaccinations lacked trust in vaccines and health services, and that this was shaped by negative experiences of healthcare and discrimination in Romania and the UK. Discrimination and marginalisation have been highlighted elsewhere in the literature as barriers that appear particularly ‘distinct’ and heightened in Roma and Romanian communities [31–33]. Discrimination has been directed at Romanian communities in the UK who, as put by Fox, Moroşanu and Szilassy, ‘have borne the brunt of public anxiety over Eastern European immigration’ [34]. Romanians and Roma have been portrayed as communities to fear, and to hold in contempt [34, 35]. The impact of Brexit has also contributed to this, with Roma particularly uncertain about their position in the UK [36].

Although concerns around vaccine safety and importance were reported these appeared to be less prominent factors affecting vaccination uptake. Uptake was therefore linked to the broader inequalities experienced by the communities [37].

The CMs that declined vaccination in this study demonstrated vaccine hesitancy, and their views were altered through gaining a trusting relationship with healthcare professionals. Trust can only be achieved through effective healthcare professional and provider engagement, which is reliant on the development and maintenance of links with communities. Developing trust largely relies on the development of mutual awareness and understanding between providers and community members, and the overcoming of communication barriers [33].

To promote vaccination uptake amongst the communities, new approaches must be adopted by providers that move away from traditional one-size-fits-all efforts. This will include a need to consider the use of outreach approaches and drop-in services. A shift towards mandating vaccination, which has been raised in the UK in relation to MMR, is unlikely to help in developing the trust needed for effective community engagement. We recommend that efforts are placed in engaging with communities, ensuring the availability of credible vaccine information and promoting the accessibility of health services and vaccinations.

## Strengths and limitations

In conducting this research, we have reflected on the heterogeneity within Romanian and Roma communities, such as differences in linguistic skills and cultural backgrounds, and the importance of considering the influence of many variables affecting vaccination uptake. This is necessary to ensure that nationality and ethnicity are not simply used as a proxy for other variables [38].

In this study, we sought to identify and understand factors affecting vaccination uptake amongst Romanian and Roma Romanian communities in 3 areas affected by measles outbreaks in 2017–18. In speaking to providers, we gained insight into some of the main factors affecting uptake amongst those most at risk of vaccine-preventable disease.

The small number of CMs that we interviewed in this study were open, confident and mostly well-linked with health services and the community. Our participants were therefore not representative of less well-connected women in the communities; however, speaking with these women has provided valuable insight into some of the barriers to vaccination uptake faced by Romanian and Roma communities. We therefore anticipate that less connected women in the communities may experience greater barriers to vaccination and health services access.

In interviewing providers, it may have been that some of their comments were based more on stereotypes and preconceived ideas about the communities, rather than real life experiences. Providers acknowledged having few links and a lack of understanding of the communities.

## Conclusion

We found that access and acceptance were dominant barriers to vaccination amongst the Roma and Romanian communities in this study, and that the development of trust from face to face contact between communities and providers is key to promoting vaccination. To establish and maintain trust providers must find ways to connect with and develop a greater understanding of the communities they serve. Better provider-service user relationships are essential to promote not only vaccination but also to reduce inequalities in health service access more broadly.

## Data Availability

All data relevant to the study are included in the article.

## Abbreviations

CM: Community member
DNA: Did not attend
GP: General practice
GRT: Gypsy, Roma and Traveller
MMR: Measles, mumps and rubella
NHS: National Health Service
PHE: Public Health England
UK: United Kingdom
WHO: World Health Organisation
